# The Effect of Angulation and Scan Body Position on Scans for Implant‐Treated Edentulism: A Clinical Simulation Study

**DOI:** 10.1111/cid.70001

**Published:** 2025-03-03

**Authors:** Georgia Vasileiadi, Evangelos Ximinis, Katia Sarafidou, Theodora Slini, Fotis Gogomitros, George Andreas Athanasiadis, Olga Naka, Alexandros Tsouknidas, Athina Bakopoulou, Maria Kokoti

**Affiliations:** ^1^ Department of Prosthodontics, School of Dentistry Aristotle University of Thessaloniki (AUTh) Thessaloniki Greece; ^2^ Department of Mechanical Engineering, School of Engineering Aristotle University of Thessaloniki (AUTh) Thessaloniki Greece; ^3^ Laboratory of Biomaterials and Computational Biomechanics, Department of Mechanical Engineering University of Western Macedonia Kozani Greece

**Keywords:** acquisition of digital impression, all‐on‐6 dental implant prostheses, implant angulation, intraoral scanners, scan body, scanning protocol

## Abstract

**Introduction:**

The acquisition of digital impressions has become an integral part of clinical dentistry. The purpose of the present clinical simulation study was to evaluate the accuracy of digital impressions for maxillary full‐arch implant‐supported prostheses using two modern intraoral scanners with different acquisition technologies.

**Material and Methods:**

Two models of edentulous maxilla, with six implants at positions #16,14,12,22,24,26 (FDI World Dental Federation System, ISO 3950) or #3,5,7,10,12,14 (Universal Numbering system) were digitally designed, and 3D‐printed in resin material (Asiga DentaMODEL, Australia). In the first scenario, all implants were parallelized, while in the second, implants #12/#7 and #22/#10 had a 20° angulation buccally, while implants #16/#3 and #26/#14 20° angulation distally. The models were scanned with two different intraoral scanners, Trios3 (3Shape, Denmark) and CS3600 (Carestream Dental, USA). Linear (x, y, z axes—top point) and angular deviations (x, y, z axes—Δφ) were assessed. Statistical analysis was performed using Kolmogorov–Smirnov tests (significance at *p* < 0.05).

**Results:**

Implant angulation showed a significant impact on accuracy, while the two scanners showed statistically significant differences. CS3600 demonstrated superior trueness, while Trios3 superior precision in both clinical scenarios. In the first clinical scenario a predominant occurrence of angular deviations was observed, while in the second scenario both angular and linear deviations were recorded. Scan body position also influenced scanning outcomes, with the last scan body captured demonstrating higher deviations.

**Conclusion:**

Both scanners provided acceptable accuracy in the acquisition of digital impressions. Implant angulation and scan body position significantly affected trueness and precision. Clinicians should carefully consider implant angulations in full‐arch implant restorations, as well as the scanning protocol.

## 1 Introduction

In recent years, digital implant technology has revolutionized the fabrication of dental restorations, offering a more efficient and accurate alternative to traditional techniques. Digital impressions, captured using intraoral scanners (IOS), provide a precise digital twin of the oral cavity. This virtual model, stored as an STL file (Standard Tessellation Language), serves as the foundation for digitally designing and manufacturing prosthetic restorations via modern Computer‐Aided Design/Computer‐Aided Manufacturing (CAD/CAM) techniques.

Passive fit is critical for the long‐term success of full‐arch implant‐supported fixed prostheses [1, 2]. A misfit between the implant abutment and the suprastructure would be detrimental for the long‐term survival of the prosthesis, as stress generation due to the misfit may lead to technical complications. An important prerequisite for achieving sufficient fit is the accurate conduction of impression‐making and transfer of the implant position to the final working cast. In a conventional workflow, the implant position is transferred to a stone cast through elastomeric impression materials. The accuracy of conventional implant impressions is highly affected by the type of impression material and technique, the surface modifications of impression copings, the magnitude of angulations in implant positions concerning the horizontal crestal plane, and the depth in which the implants are placed supracrestally in the patient's bone [3]. On the other hand, in a digital workflow, intraoral scan bodies (ISbs) are being positioned onto the implants to accurately transfer implant position to the virtual model. Factors affecting the accuracy of digitally acquired impressions may be the type of scanning device, the operator's experience, the implant angulation, the design characteristics of scan bodies, the number of implants, and the inter‐implant distance, as well as the implants' distribution across the arch [4, 5].

There are several IOS devices available in the dental market, while new scanners are being continuously introduced, bearing different features and new specifications, overall rendering the choice of the most appropriate device quite complicated for the dental clinicians and specialists in prosthodontics that wish to switch their daily practice to a fully digital mode. While speed, tip size, and price, also play a crucial role in device selection, accuracy remains of the most paramount importance [6, 7, 8]. The accuracy of different intraoral scanners has been evaluated in several studies, demonstrating variable levels of performance among digital devices [5].

The accuracy is defined by both “precision” that is, the consistency of replicate measurements, and “trueness”, that is, the conformance to the actual dimensions (ISO 5725‐1) [9]. Both precision and trueness are critical parameters in the digital implant dentistry workflow [5, 7]. Precision refers to how close repeated measurements of the same object are to each other, and can be evaluated by superimposing different scans of the same object, obtained by the same IOS device. Trueness depicts how far the measurement deviates from the actual dimensions of the measured object and can be evaluated by superimposing scans of the same object obtained by a reference scanner and different IOS devices [9]. To evaluate accuracy, it is mandatory to have a reference scanner with error tending to zero, such as a coordinate measuring machine or an industrial optical scanner with accuracy < 5 μm [10, 11]. After obtaining virtual models, scans are evaluated using reverse engineering software, to mathematically determine linear and angular deviations.

Information provided by the literature so far is mostly restricted to the accuracy of fit of single‐unit or short‐span implant‐supported restorations employing IOS for digitally capturing the implant position and showing that the observed deviations on the virtual model remain within acceptable clinical standards, that is, < 120 μm [4, 12, 13]. However, implant‐supported fixed prosthesis to restore complete edentulism complicates scanning procedures and affects the quality of the obtained scans. In cases where multiple implants are inserted, no consensus has been reached so far regarding the accuracy of IOS devices and their reliability compared with conventional impression methods.

The most common treatment protocol for restoring complete edentulism by means of a full‐arch fixed prosthesis, includes placement of six implants in the maxilla, and five implants in the mandible [14]. Implant distribution seems to contribute to the long‐term survival of full‐arch restorations, with a symmetrical anteroposterior arrangement reducing the cantilever length and allowing better stress distribution [15]. To secure an even stress distribution in the maxilla, the optimal sites for placing implants are considered the areas of the first molars, the first premolars and the lateral incisors, or alternatively the second premolars, the canines and the central incisors [16]. Implant angulation may also influence digital impression accuracy.

Certain investigators have reported no significant impact of implant angulations in full‐arch implant prosthesis fit in complete edentulous arches [17, 18], while others have reported an association between implant angulation and linear deviations [19, 20].

Based on the above, it was the purpose of the present in vitro study was to evaluate the accuracy of digital impressions for maxillary full‐arch implant‐supported fixed prostheses simulating two different clinical scenarios (with or without implant angulation) and employing two different IOS devices. The null hypothesis was that there would be no difference in the accuracy of the digital impressions between the two IOS devices, and/or with variable angulations among implants, in the full‐arch implant‐supported prosthesis of each clinical scenario.

## 2 Materials and Methods

**FIGURE 1:**
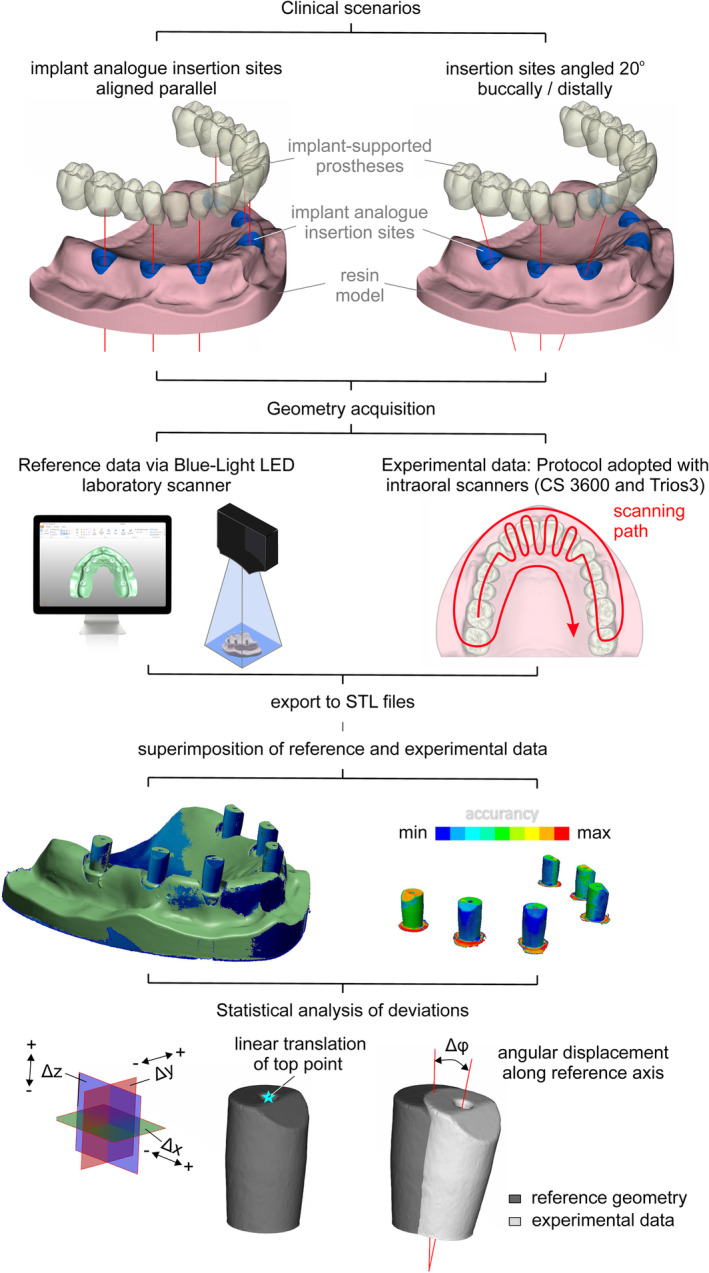
Flowchart of the study.

**FIGURE 2:**
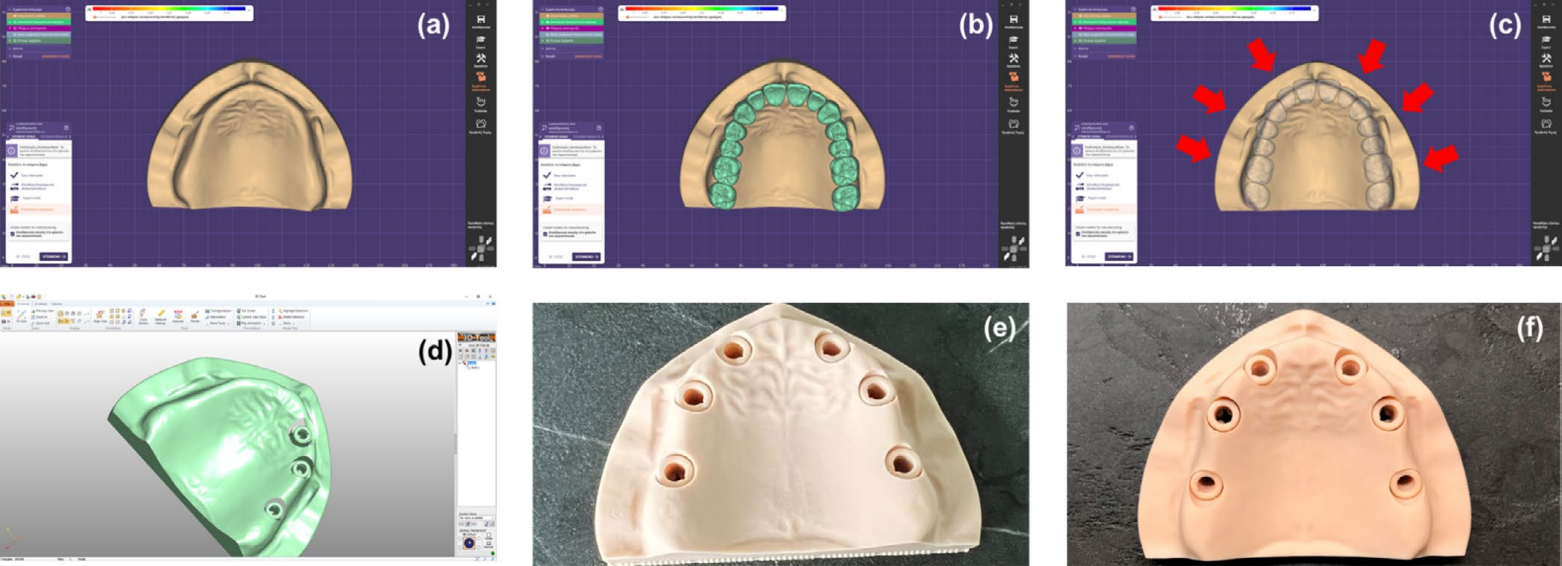
Design and fabrication of the study models. (a) The polymer model of edentulous maxilla. (b) Digital diagnostic wax up. (c) Determination of the optimal implant placement positions. (d) Digital design of edentulous maxilla models (ANSA, Beta CAE Systems). (e) The printed model 1 (SLA, Asiga Max 3d printer). (f) The printed model 2 (SLA, Asiga Max 3d printer).

The digital and technical steps of the present investigation are depicted as a flowchart in Figure 1. A polymer model of an edentulous maxilla (KaVo, Biberach, Germany) was coated with a nonfluorescent bright white developer (SKD‐S2), to reduce surface reflectivity [21] and suppress systematic and random errors during the digitization of the model into an STL file via a VTOP300T laboratory scanner (Visentech, Tianjin, China). A digital diagnostic wax up (Exocad DentalCAD; Exocad GmbH, Darmstadt, Germany) was then performed to determine the optimal positions for implant insertion. Two 3D edentulous maxilla models were created (using ANSA software from Beta CAE Systems) [ANSA, Beta CAE systems Inc., USA], with six implant insertion sites, coinciding with the positions of the lateral incisors, first premolars and first molars [#16,14,12,22,24,26 (FDI notation, ISO 3950) or #3,5,7,10,12,14 (Universal Numbering system)]. Subsequently, an implant analog (ElosMedtech, Gothenburg, Sweden) was scanned, and its external surface was integrated into the models at the designated implant positions. Finally, the two maxillary models with the six integrated implant analogs at the positions #16,14,12,22,24,26 (FDI notation, ISO 3950) or #3,5,7,10,12,14 (Universal Numbering system) were 3D printed (SLA stereolithography printer, Asiga, Max 3d printer, Sydney, Australia) in resin material (Asiga DentaMODEL, Asiga, Sydney, Australia). These 3D‐printed models were used to simulate two different clinical scenarios (Figure 2).

The two maxillary models, representing the two clinical scenarios, were 3D printed (SLA stereolithography printer, Asiga, Max 3d printer, Sydney, Australia) in resin material (AsigaDentaMODEL, Asiga, Sydney, Australia). The Asiga Max (Asiga, Sydney, Australia), operates on digital light processing (DLP) technology, utilizing a dual wavelength of 385/405 nm with a 62‐μm pixel size. Additionally, it features a curing chamber, the Asiga Flash (Asiga, Sydney, Australia), which exposes printed models to 405 nm light for curing, albeit without heating. In our study, the two models were printed utilizing DentaMODEL resin from Asiga Australia. After fabrication, the two models were ultrasonically cleaned in 98% isopropyl alcohol for 10 min (5 min of prewash and 5 min of post‐wash), according to the manufacturer's guidelines. Subsequently, the models were left to air‐dry for a minimum of 10 min to ensure the complete evaporation of the residual solvent before undergoing a curing process in the Asiga Flash unit for 20 min. Post‐curing is a UV‐light treatment to ensure that DentaMODEL printed parts obtain optimal polymer conversion. Through this procedure the residual monomer is reduced to a minimum and the required mechanical properties are obtained. The AsigaDentaMODEL resin, exhibits a polymerization shrinkage of approximately 1%. This low shrinkage level helps ensure the accuracy and dimensional stability of printed dental models. The two printed models simulating two different clinical scenarios were made from the same material (AsigaDentaMODEL) under the same conditions, in order to minimize the polymerization shrinkage.

**FIGURE 3:**
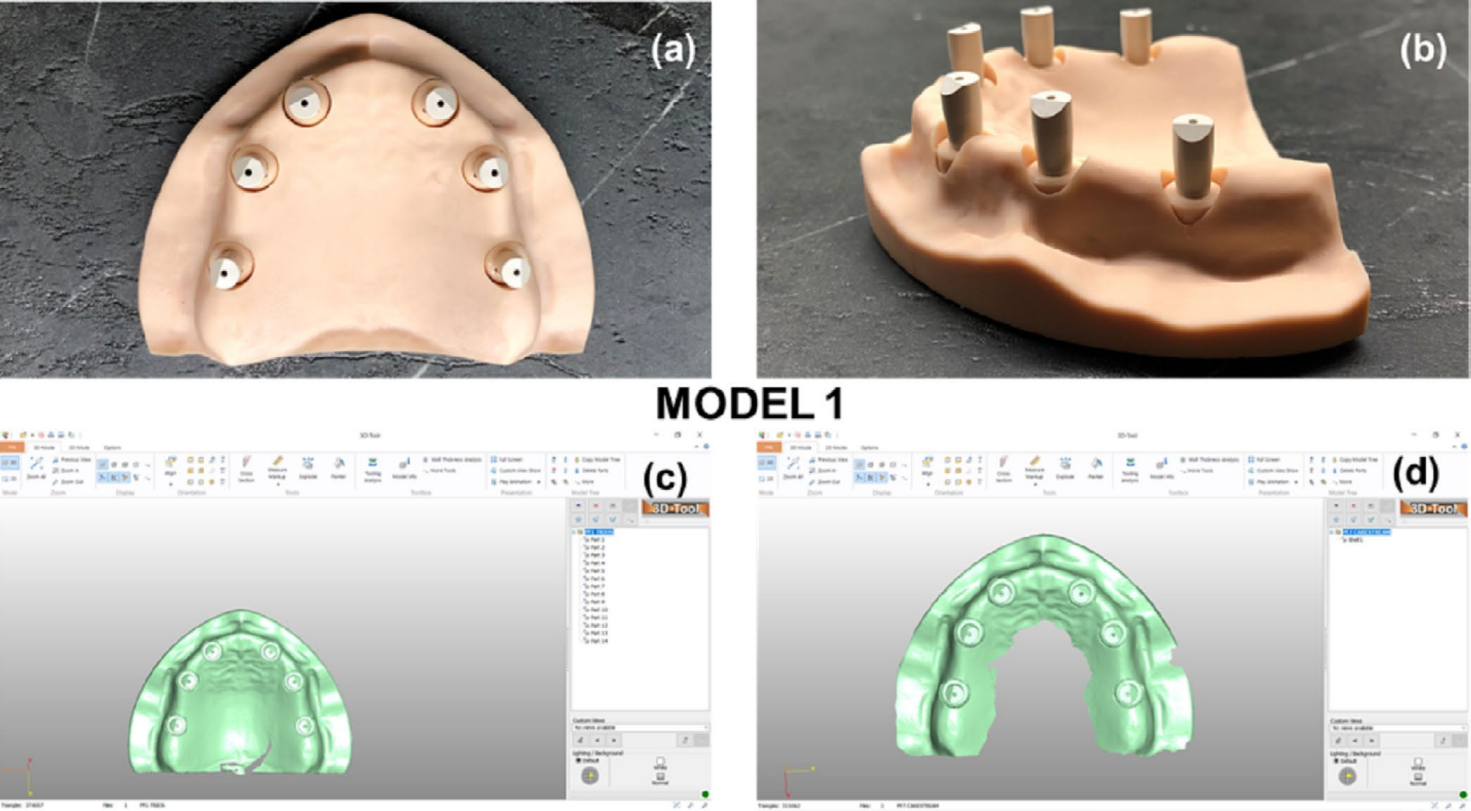
First clinical scenario (Model 1). (a) Model 1 with six PEEK scanbodies attached to the abutments (vertical view). (b) Model 1 with six PEEK scanbodies attached to the abutments (side view). (c) Scan of model 1 with Trios3—3Shape. (d) Scan of model 1 with Carestream 3600—Carestream.

**FIGURE 4:**
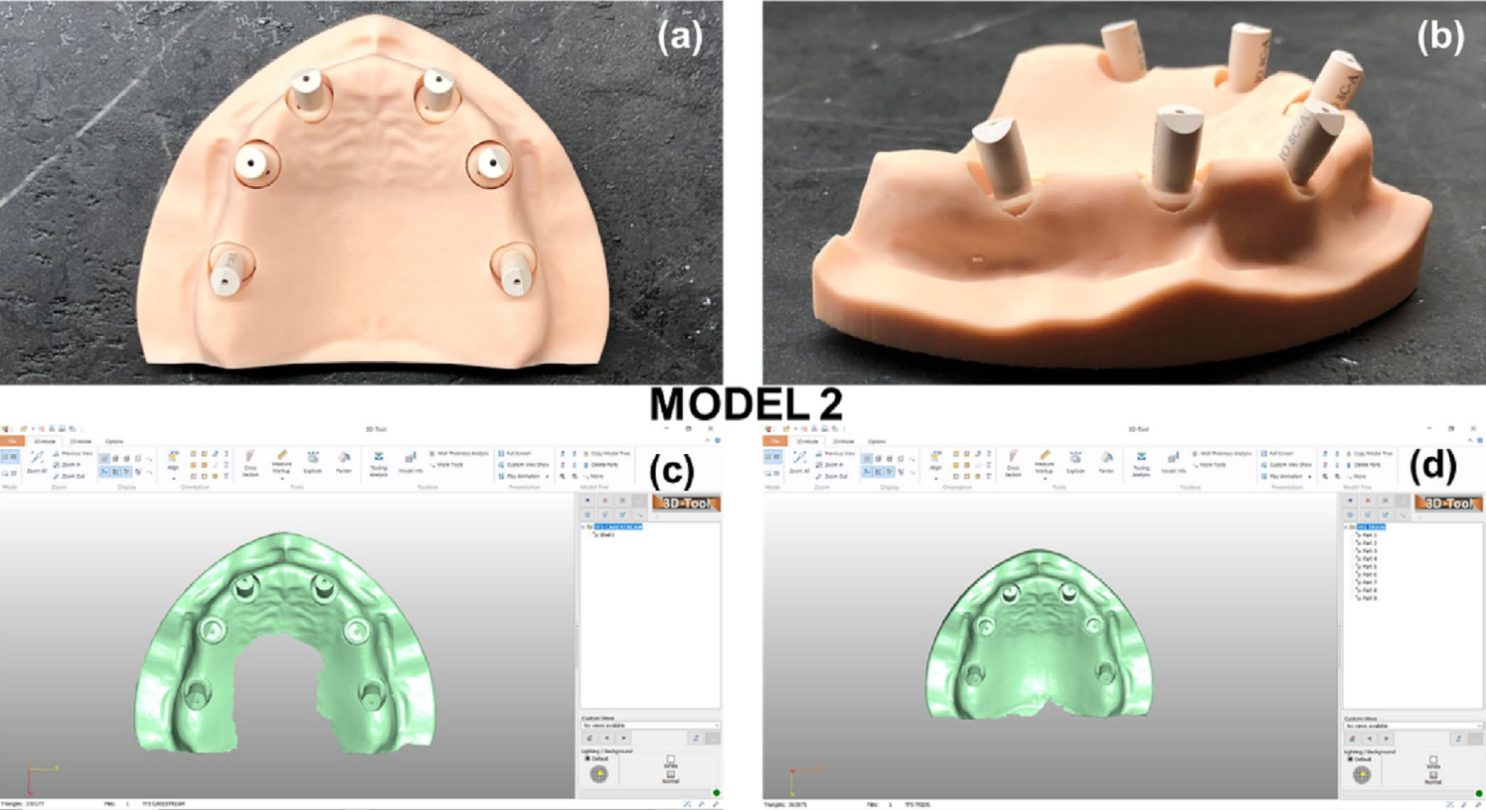
Second clinical scenario (Model 2). (a) Model 2 with six PEEK scanbodies attached to the abutments (vertical view). (b) Model 2 with six PEEK scanbodies attached to the abutments (side view). (c) Scan of model 2 with Trios3—3Shape. (d) Scan of model 2 with Carestream 3600—Carestream.

In the first clinical scenario (model 1), all implant analogs were aligned parallel to each other and perpendicular to the occlusal plane (Figure 3). In the second clinical scenario (model 2), implant analogs #12/(7) and #22/(10) were angled 20° buccally, while implant analogs #16/(3) and #26/(14) were angled 20° distally. The latter recapitulates common difficulties arising in clinical cases, where implant placement with such angulations is conducted due to bone resorption in the anterior region, and limited bone height in the posterior region, respectively. Implant analogs #14 and #24 were inserted parallel to each other and perpendicular to the occlusal plane (Figure 4). The positions and orientations of the implant analogs were digitally designed before 3D‐printing. All implant analogs were submerged 2 mm below the surface in the maxillary model, and balance‐base abutments (ElosMedtech, Gothenburg, Sweden) were attached and fastened to the implant analogs with a torque of 25 Nm, according to the manufacturer recommendations.

Six polyether‐ether‐ketone (PEEK) scan bodies (ElosMedtech, Gothenburg, Sweden) were, then, attached to the abutments of model 1 and tightened manually with a torque of approximately 10 Nm. Model 1 was scanned 10 times using each intraoral scanner [10 scans with CS 3600 (Carestream Dental, Rochester, NY, USA), 10 scans with Trios3 (3Shape, Copenhagen, Denmark)], following the manufacturer's instructions. Scanning began with the occlusal surfaces, employing an antiprismatic polygon pattern (i.e., zig‐zag like path), in the anterior area. Subsequently, the buccal surfaces were captured, followed by the palatal ones. The starting point was always the most distal scan body (#16/(3)). The scanner's camera was aligned parallel to the occlusal plane and positioned as close as possible to the maxillary surfaces. Each scanner was pre‐calibrated for 3D and color accuracy, according to the manufacturer's recommendations, prior to switching experimental groups. The six scan bodies were then unfastened from model 1 and tightened manually with a torque of approximately 10 Nm onto the abutments of model 2. The same scanning protocol as described for model 1 was followed for model 2.

To establish reference scans, the models of both clinical scenarios were initially scanned using a Blue‐Light LED laboratory scanner (Vinyl High Resolution, Smart Optics, Oslo, Norway), presenting an accuracy of 4 μm (ISO 12836). The laboratory scanner was calibrated in accordance with the manufacturer's instructions prior to scanning. The reference scans were exported as STL files. All digital scanning procedures were conducted by a single operator, after calibration with 30 trial digital scans for each IOS system, as instructed by the manufacturer. From each clinical scenario, 10 scans were acquired for each intraoral scanner and exported as .STL files. These scans were then imported into inspection software (Autodesk Meshmixer, SolidWorks 2024, USA) for evaluation of trueness and precision.

## 3 Data Processing and Evaluation of Scanning Accuracy

The digital analysis of the scans was conducted in the following manner. The scans were categorized into folders based on their respective groups (scenario 1–scenario 2), and the IOS device (Carestream–Trios). The analysis process commenced with aligning the orientation of each group of scans. Ensuring the accuracy of this alignment was crucial for evaluating the precision and reproducibility of the measurements, and the latter was accomplished in two consecutive steps.

- Manually: The reference scanner files were used as position/base orientation for manual alignment in the Meshmixer software. This process was repeated for each measurement separately, while all files were saved in a new folder to preserve their new orientation (position relative to the reference coordinates).
- Automatically: For a confirmation of the correctness of the orientation, the scans were automatically aligned using an automated alignment command in the software SolidWorks 2024 (SolidWorks Corp., MA, USA) This two‐step approach ensured that the scans were aligned accurately for subsequent analysis.

Next, the files of each group were imported in the interface of SolidWorks 2024 (SolidWorks Corp., MA, USA). The software was employed to locate the coordinates (Top, base) of the scan bodies. To assess linear deviations, a separate evaluation of the 3 coordinates (x, y, z) of the top point of each scan body was conducted (i.e., starting with position 16/(3), then moving to position 14/(5) etc.). To assess angular deviations, a separate evaluation of the 3 coordinates (x, y, z) of the angle (Δφ) of each scan body was performed (i.e., beginning with position 16/(3), then moving to position 14/(5) etc.).

## 4 Statistical Analysis

Statistical analysis was conducted using the SPSS 29 statistical software (IBM, SPSS Inc., Chicago, IL, USA). To comprehensively assess the data set, descriptive statistics indicators, including mean and standard deviation, were initially calculated. Subsequently, both Kolmogorov–Smirnov and Shapiro–Wilk tests of normality were conducted, to evaluate and measure the quality and fitness of the data set. In addition, Q‐Q plots displayed the accuracy of fit. Furthermore, non‐parametric Kruskal‐Wallis and Mann Whitney tests were employed to detect differences in distribution between the various methodologies (desks). The level of significance was set at 0.05.

## 5 Results

**FIGURE 5:**
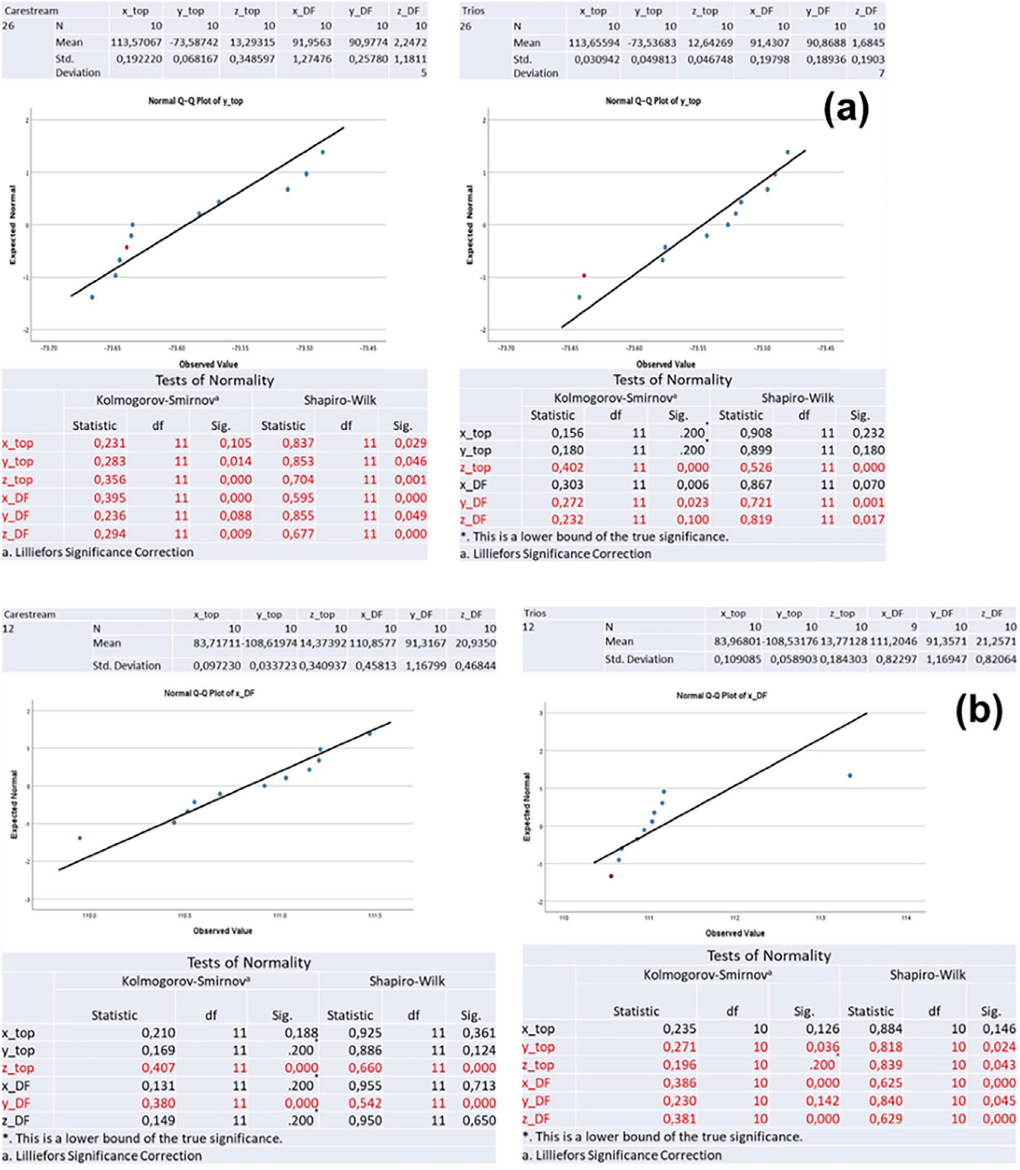
Carestream versus Trios. Statistical significances are given in red color. (a) Position #26—y_top—first clinical scenario—Carestream versus Trios. Statistical significances are given in red color. (b) Position #12—x_Δφ—second clinical scenario—Carestream vs. Trios. Statistical significances are given in red color.

In both clinical scenarios the yielded scans provided an accuracy within the clinically acceptable limits [22, 23]. Tables 1, 2, 3, 4, 5, 6 and Figure 5 depict the results of the statistical analysis, which revealed a statistically significant impact of the scanner type and implant angulation on the accuracy of digital impressions. These findings led to the rejection of the null hypothesis. Particularly, the scanner type, the implant angulation and the implant position had a significant influence on both angular and linear deviation measurements (*p* < 0.05).

**TABLE 1:** Evaluation of “trueness” in acquisition in the first case scenario. The implant positions given in the table (as per FDI World Dental Federation System (ISO 3950), and in parenthesis the Universal Numbering System) showed statistically significant differences among control and test scans.

Scanner	Implant position	Evaluation	Coordinate axis	Test of normality—Shapiro–Wilk		
				Statistic	df	sig
CS 3600	16 (3)	Top point	z	0.806	11	0.011
CS 3600	14 (5)	Top point	z	0.719	11	0.001
CS 3600	12 (7)	Top point	z	0.755	11	0.002
CS 3600	22 (10)	Top point	z	0.753	11	0.002
CS 3600	24 (12)	Top point	z	0.704	11	0,001
CS 3600	26 (14)	Top point	x	0.837	11	0.029
CS 3600	26 (14)	Top point	y	0.853	11	0.046
CS 3600	26 (14)	Top point	z	0.704	11	0.001
CS 3600	26 (14)	Δφ	x	0.595	11	0.000
CS 3600	26 (14)	Δφ	y	0.855	11	0.049
CS 3600	26 (14)	Δφ	z	0.677	11	0.000
TRIOS 3	16 (3)	Top point	z	0.724	11	0.001
TRIOS 3	14 (5)	Top point	z	0.657	11	0.000
TRIOS 3	14 (5)	Δφ	x	0.811	11	0.013
TRIOS 3	12 (7)	Top point	x	0.505	11	0.000
TRIOS 3	12 (7)	Top point	z	0.707	11	0.001
TRIOS 3	22 (10)	Top point	z	0.620	11	0.000
TRIOS 3	22 (10)	Δφ	x	0.828	11	0.022
TRIOS 3	22 (10)	Δφ	z	0.672	11	0.000
TRIOS 3	24 (12)	Top point	z	0.715	11	0.001
TRIOS 3	26 (14)	Top point	z	0.526	11	0.000
TRIOS 3	26 (14)	Δφ	y	0.721	11	0.001
TRIOS 3	26 (14)	Δφ	z	0.819	11	0.017

*Note:* Top point: Evaluation of linear deviation. Δφ: evaluation of angular deviation.

**TABLE 2:** Evaluation of “trueness” in acquisition in the second case scenario. The implant positions given in the table (as per FDI World Dental Federation System (ISO 3950), and in parenthesis the Universal Numbering System) showed statistically significant differences among control and test scans.

Scanner	Implant position	Evaluation	Coordinate axis	Test of normality—Shapiro–Wilk		
				Statistic	df	sig
CS 3600	16 (3)	Top point	z	0.735	11	0.001
CS 3600	14 (5)	Top point	x	0.838	11	0.029
CS 3600	14 (5)	Top point	z	0.511	11	0.000
CS 3600	12 (7)	Top point	z	0.660	11	0.000
CS 3600	12 (7)	Δφ	y	0.542	11	0.000
CS 3600	22 (10)	Top point	z	0.686	11	0.000
CS 3600	24 (12)	Top point	z	0.448	11	0.000
CS 3600	26 (14)	Top point	x	0.849	11	0.042
CS 3600	26 (14)	Top point	y	0.851	11	0.044
CS 3600	26 (14)	Top point	z	0.651	11	0.000
TRIOS 3	14 (5)	Top point	z	0.813	11	0.014
TRIOS 3	14 (5)	Δφ	y	0.734	11	0.001
TRIOS 3	12 (7)	Top point	y	0.818	10	0.024
TRIOS 3	12 (7)	Top point	z	0.839	10	0.043
TRIOS 3	12 (7)	Δφ	x	0.625	10	0.000
TRIOS 3	12 (7)	Δφ	y	0.840	10	0.045
TRIOS 3	12 (7)	Δφ	z	0.629	10	0.000
TRIOS 3	22 (10)	Top point	x	0.814	11	0.014
TRIOS 3	22 (10)	Top point	z	0.622	11	0.000
TRIOS 3	22 (10)	Δφ	x	0.725	11	0.001
TRIOS 3	22 (10)	Δφ	y	0.741	11	0.002
TRIOS 3	22 (10)	Δφ	z	0.770	11	0.004
TRIOS 3	24 (12)	Top point	x	0.424	10	0.000
TRIOS 3	24 (12)	Top point	z	0.417	10	0.000
TRIOS 3	24 (12)	Δφ	x	0.497	10	0.000
TRIOS 3	24 (12)	Δφ	y	0.743	10	0.003
TRIOS 3	24 (12)	Δφ	z	0.839	10	0.043
TRIOS 3	26 (14)	Top point	x	0.745	9	0.005
TRIOS 3	26 (14)	Top point	z	0.805	9	0.023

*Note:* Top point: Evaluation of linear deviation. Δφ: evaluation of angular deviation.

**TABLE 3:** Evaluation of “precision” in acquisition in the first case scenario employing the Carestream IOS. Mean and standard deviation for each scan body position in all axes (x, y, z coordinates)—top point and Δφ—10 scans. The implant positions are provided as per FDI World Dental Federation System (ISO 3950) and in parenthesis the Universal Numbering System.

Model	Implant position		Top point coordinate axis			Δφ coordinate axis		
			Top point (x)	Top point (y)	Top point (z)	Δφ (x)	Δφ (y)	Δφ (z)
Model 1	12 (7)	*N*	10	10	10	9	10	10
		Mean	86.00583	−108.22159	14.92710	90.6227	91.1507	1.3288
		Std. Deviation	0.056203	0.033184	0.377276*	0.17607	0.15 000	0.16441
	14 (5)	*N*	10	10	10	10	10	10
		Mean	97.08008	−116.96947	14.66803	90.4250	90.5770	0.7724
		Std. Deviation	0.051425	0.042211	0.412834*	0.25481	0.46 858	0.43835
	16 (3)	*N*	10	10	10	10	10	10
		Mean	113.58100	−121.03216	12.39446	90.6595	90.5704	0.9680
		Std. Deviation	0.085265	0.106875*	0.421543*	0.37645	0.60013	0.55269
	22 (10)	*N*	10	10	10	10	10	10
		Mean	86.04710	−86.44881	15.55914	90.9391	90.7255	1.2259
		Std. Deviation	0.062399	0.047170	0.296996*	0.29072	0.27608	0.23640
	24 (12)	*N*	10	10	10	10	10	10
		Mean	97.14442	−77.72756	15.53691	90.8776	91.1570	1.5016
		Std. Deviation	0.057129	0.042434	0.345280*	0.30946	0.33641	0.21500
	26 (14)	*N*	10	10	10	10	10	10
		Mean	113.57067	−73.58742	13.29315	91.9563	90.9774	2.2472
		Std. Deviation	0.192220	0.068167	0.348597*	1.27476	0.25780	1.18115

* Significant (*p* < 0.05).

**TABLE 4:** Evaluation of “precision” in acquisition in the first case scenario employing the Trios IOS. Mean and standard deviation for each scan body position in all axes (x, y, z coordinates)—top point and Δφ—10 scans. The implant positions are provided as per FDI World Dental Federation System (ISO 3950) and in parenthesis the Universal Numbering System.

Model	Implant position		Top point coordinate axis		Δφ coordinate axis			
			Top point (x)	Top point (y)	Top point (z)	Δφ (x)	Δφ (y)	Δφ (z)
Model 1	12 (7)	*N*	10	10	10	10	10	10
		Mean	86.06580	−108.15987	14.49459	90.9934	91.0420	1.4497
		Std. Deviation	0.096538*	0.025326	0.273907*	0.25246	0.20626	0.27191
	14 (5)	*N*	10	10	10	10	10	10
		Mean	97.07945	−116.93659	14.22453	90.2265	90.5664	0.7075
		Std. Deviation	0.029474	0.039950	0.083114	0.43645	0.31224	0.38117
	16 (3)	*N*	10	10	10	10	10	10
		Mean	113.60605	−121.11908	11.99673	90.7027	90.3331	0.7915
		Std. Deviation	0.019901	0.036743	0.277759*	0.16579	0.18040	0.18953
	22 (10)	*N*	10	10	10	10	10	10
		Mean	86.05754	−86.44327	14.60595	91.1903	90.5638	1.3386
		Std. Deviation	0.052744	0.031816	0.812918*	0.39 926	0.23990	0.39211*
	24 (12)	*N*	10	10	10	10	10	10
		Mean	97.10014	−77.69 334	14.89528	91.0106	90.7729	1.2901
		Std. Deviation	0.037309	0.024238	0.198446*	0.16 202	0.20184*	0.12966
	26 (14)	*N*	10	10	10	10	10	10
		Mean	113.65594	−73.53683	12.64269	91.4307	90.8688	1.6845
		Std. Deviation	0.030942	0.049813	0.046748	0.19798*	0.18936	0.19037

* Significant (*p* < 0.05).

**TABLE 5:** Evaluation of “precision” in acquisition in the second case scenario employing the Carestream IOS. Mean and standard deviation for each scan body position in all axes (x, y, z coordinates)—top point and Δφ—10 scans. The implant positions are provided as per FDI World Dental Federation System (ISO 3950) and in parenthesis the Universal Numbering System.

Model	Implant position		Top point coordinate axis		Δφ coordinate axis			
			Top point (x)	Top point (y)	Top point (z)	Δφ (x)	Δφ (y)	Δφ (z)
Model 2	12 (7)	*N*	10	10	10	10	10	10
		Mean	83.71711	−108.61974	14.37392	110.8577	91.3167	20.9350
		Std. Deviation	0.097230	0.033723	0.340937*	0.45813	1.16799*	0.46844
	14 (5)	*N*	10	10	10	10	10	10
		Mean	97.91240	−116.82917	14.85972	90.5234	90.6106	0.8574
		Std. Deviation	0.054178	0.087902	0.248952*	0.28609	0.38241	0.36094
	16 (3)	*N*	10	10	10	10	10	10
		Mean	117.35351	−120.17898	11.92563	70.5495	90.1897	19.4577
		Std. Deviation	0.161087	0.127 532	0.368466*	0.24354	0.49102	0.24256
	22 (10)	*N*	10	10	10	10	10	10
		Mean	82.87502	−86.85258	14.71008	110.9695	91.7224	21.0487
		Std. Deviation	0.083180	0.074871	0.326242*	0.45971	0.28572	0.46095
	24 (12)	*N*	10	10	10	10	10	10
		Mean	96.18572	−77.44910	15.48795	91.3226	90.9670	1.6791
		Std. Deviation	0.026490	0.098671	0.210812*	0.27100	0.35303	0.22083
	26 (14)	*N*	10	10	10	10	10	10
		Mean	115.58866	−7259272	12.57812	70.8109	90.4407	19.2033
		Std. Deviation	0.120125*	0.115124*	0.328910*	0.25343	0.58975*	0.24988

* Significant (*p* < 0.05).

**TABLE 6:** Evaluation of “precision” in acquisition in the second case scenario employing the Trios IOS. Mean and standard deviation for each scan body position in all axes (x, y, z coordinates)—top point and Δφ—10 scans. The implant positions are provided as per FDI World Dental Federation System (ISO 3950) and in parenthesis the Universal Numbering System.

Model	Implant position		Top point coordinate axis			Δφ coordinate axis		
			Top point (x)	Top point (y)	Top point (z)	Δφ (x)	Δφ (y)	Δφ (z)
Model 2	12 (7)	*N*	10	10	10	9	10	10
		Mean	83.96801	−108.53176	13.77128	111.2046	913571	21.2571
		Std. Deviation	0.109085	0.058903*	0.184303	0.82297*	1.16947*	0.82064*
	14 (5)	*N*	10	10	10	10	10	10
		Mean	97.88230	−116.71377	14.14436	90.5858	90.5496	0.8914
		Std. Deviation	0.015153	0.029841	0.167552	0.11316	0.41575*	0.14051
	16 (3)	*N*	10	10	10	10	10	9
		Mean	117.14911	−12022588	11.45472	70.9435	90.3724	19.0836
		Std. Deviation	0.091383	0.114331	0.289640	0.29392	0.59050*	0.31300
	22 (10)	*N*	10	10	10	10	10	10
		Mean	83.13890	−86.85188	14.09946	110.9369	91.5006	20.9961
		Std. Deviation	0.049037	0.019797	0.079021	0.14884*	0.13923	0.15501
	24 (12)	*N*	10	10	10	10	10	9
		Mean	96.17375	−77.47699	14.71080	91.4182	90.6620	1.5731
		Std. Deviation	0.012119	0.023881	0.062162	0.11053	0.13219	0.09722
	26 (14)	*N*	10	10	10	9	9	10
		Mean	115.28998	−72.59365	11.95079	70.8730	90.2742	19.1226
		Std. Deviation	0043356	0068593	0.122191	0.08972	0.23913	0.08803

* Significant (*p* < 0.05).

Results regarding the “trueness” of the acquisition of the desk scan and the repeated scans performed employing the CS 3600 IOS and the TRIOS 3 IOS devices in the first case scenario are shown in Table 1. According to Shapiro–Wilk test, in the first clinical scenario (Model 1), when utilizing the Carestream 3600 scanner, the scan body at position #26/(14) always exhibited statistically significant deviations in both linear and angular measurements across all axes [Top point (x, y, z), Δφ (x, y, z)] (*p* < 0.05). Based on the Shapiro–Wilk test in the first clinical scenario (Model 1), when utilizing the Trios 3 scanner there were statistically significant differences (*p <* 0.05) reported for the positions #12/(7) (linear deviation—Top point), #14/5 (angular deviation—Δφ), #22/(10) (angular deviation—Δφ), and #26/(14) (angular deviation—Δφ).

Accordingly, regarding the “trueness” of the acquisition of the desk scan and the repeated scans performed employing the CS 3600 IOS device and the TRIOS 3 IOS device in the second case scenario results are shown in Table 2. In the second clinical scenario (Model 2), when utilizing the Carestream 3600 scanner, the Shapiro Wilk test showed that scan bodies at the positions #14/(5) and #26/(14) exhibited significant deviations regarding the top point measurements (*p* < 0.05), while scan bodies at the position #12/(7) exhibited significant deviations regarding the Δφ measurements (*p* < 0.05). According to Shapiro–Wilk test, in the second clinical scenario (Model 2), when utilizing the Trios 3 scanner, there were statistically significant differences (*p* < 0.05) in almost all positions (#12, #14, #22, #24, #26 or 7,5,10,12,14).

In both models (Model 1–Model 2), regardless of the scanner used, a statistically significant difference (*p* < 0.05) was observed in the z‐axis coordinates of the top point for all scan body positions.

The results regarding the “precision” of the repeated scans acquired with the Carestream IOS device and the Trios IOS device in the first case scenario are shown in Tables 3 and 4, respectively. Similarly, the results regarding the “precision” of the repeated scans acquired with the Carestream IOS device and the Trios IOS device in the second case scenario are shown in Tables 5 and 6, respectively. According to the Kolmogorov–Smirnov test, in both clinical scenarios, the Carestream 3600 exhibited a non‐normal distribution in the z‐axis coordinates of the top point for all scan body positions across the repeated scans (*p* < 0.05). Notably, in the second clinical scenario, there were larger deviations in almost all axis coordinates for the scan body at position #26/(14) (*p* < 0.05) among the repeated scans. When the Trios 3 scanner was tested in the first clinical scenario, there was a non‐normal distribution in the z‐axis coordinates of the top point for almost all scan body positions across the repeated scans (*p* < 0.05), as well as in the Δφ measurements at positions #22/(10), #24/(12), and #26/(14). In the second clinical scenario, the Trios 3 exhibited statistically significant differences among the repeated scans at positions #12/(7), #14/(5), and #16/(3) regarding the Δφ measurements (*p* < 0.05). Overall, the Trios IOS device exhibited lower standard deviations among the repeated scans than the Carestream IOS device, in both case scenarios (Model 1–Model 2).

A comparison of scans acquired by the Carestream and Trios device revealed statistically significant discrepancies for specific implant positions. Representative results are illustrated in Figure 5. Figure 5a highlights the disparities in y‐axis measurements for implant #26/(14) with parallel abutments, while the Trios Q‐Q plot indicates a more normal distribution of data compared to Carestream (Figure 5a).

Figure 5b is representative of the differences in trueness between the two IOS devices for the position #12/(7) with angulated implants (Figure 5b).

## 6 Discussion

The present study comparatively evaluated the accuracy of digital impressions for maxillary full‐arch implant supported fixed prostheses on six implants simulating two different clinical scenarios, that is, with or without implant angulations. For the acquisition of digital scans two widely used intraoral scanners (Trios3—3Shape and CS 3600—Carestream) were employed. To our knowledge, these specific IOS have not been evaluated on full‐arch implant‐supported prostheses in the most challenging clinical scenarios, comprising angulated implants in both anterior and posterior areas of the maxilla. Their overall performance is, however, well established, consistently demonstrating deviations below 100 μm in most comparative studies of digital impression accuracy [22, 23, 24, 25]. The current study corroborates and further validates these findings in more complex and highly demanding restorations.

Several studies have used dental laboratory scanners instead of industrial 3D scanners to acquire reference data [23, 26, 27, 28]. Dental laboratory scanners are used to scan cast models produced from a conventional impression and create surface 3D data, which are then exported to CAD software to design the restorations. According to a recent review [29] the accuracy of industrial scanners ranges from 1 to 10 μm, whereas a laboratory scanner's accuracy ranges from 2 to 10 μm, suggesting that the accuracy of digital impressions obtained by new generation dental laboratory scanners is comparable to that of the industrial 3D scanner. Furthermore, in a recent in vitro study, where desktop scanners were compared with an industrial scanner for the evaluation of an intraoral scanner accuracy, all desktop scanners tested had the acceptable accuracy required for a complete arch‐fixed prosthesis and might be used as reference scanners for studying IOS accuracy [30]. In our study, a Vinyl high resolution laboratory scanner was used (Vinyl High Resolution, Smart Optics, Oslo, Norway). It is a new generation scanner, that features stripe light triangulation as measurement technology, a high‐performance blue light LED sensor, a 3,2 MP camera and an innovative fully automated z‐axis, which automatically moves the scan object to the correct height, improving the scanning process. Besides, it presents an accuracy of 4 μm, according to ISO 12836, and is therefore considered to be a highly reliable and accurate device. An interesting finding of the present study was the significant influence of scan body position, and the order in which the scan bodies were digitalized, on both linear and angular deviations (*p* < 0.05) of the scans compared to the real position of the implant. Scan body #26 (FDI notation, ISO 3950) or 14 (Universal Numbering system), consistently scanned last according to the scanning protocol, exhibited the highest linear and angular deviations. This result is in accordance with the findings of Gimenez et al. who reported significantly poorer results for the second quadrant scanned compared to the first one [18, 31, 32]. However, other studies have reported the opposite trend, with larger deviations observed for the first implant scanned [19, 33]. These discrepancies may arise from differences in scanning technologies, algorithm correction methods, or the type of measurements employed. Taking these findings into consideration, it appears that the scanning protocol exerts a substantial impact on digital impression quality and should therefore be carefully chosen by clinicians.

The present study rejected the null hypothesis, demonstrating a significant impact of implant angulation on scan accuracy. These findings align with Arcuri et al. [19], who concluded that implant angulation significantly influenced linear deviations, while implant position affected angular deviations. These investigators studied the influence of scan body material, position, and operator on the accuracy of an intraoral scanner for full‐arch implant impressions, and found that material and implant position significantly impacted accuracy, while no significant operator effect was observed [19].

In the first clinical scenario involving parallel implants, the Carestream 3600 scanner consistently outperformed Trios 3 in terms of trueness, with regards to both angular and linear accuracy. The highest deviations were observed for the last implant scanned (position #26 or 14) using the Carestream 3600. In contrast, with Trios 3, the first quadrant scanned exhibited superior accuracy, with errors gradually increasing from the first to the last scan body captured. This difference might be attributed to the curvature of the maxillary arch, as scanning across the midline necessitates extensive stitching, which potentially compromises accuracy and might make the scanning procedure more prone to errors [32]. Conversely, Trios 3 demonstrated superior precision in both angle and position measurements.

Similar trends emerged in the second clinical scenario, with Carestream 3600 excelling in trueness and Trios 3 taking the lead in precision. Similar to the first scenario, higher deviations were observed for the last implant scanned (#26 or 14) when using Carestream 3600. On the other hand, with Trios 3, the error gradually increased as the last scan body was approached. These findings are in accordance with the results of other in vitro studies that compared accuracy (trueness and precision) of different IOS for full arch digital impressions using 3D deviation analysis [22, 23, 24, 25]. These studies also reported the consistent superiority of Carestream 3600 concerning trueness, and of Trios 3 with regards to precision, for full‐arch digital impressions with six parallel implants. A possible explanation of the differences observed, lies on the different acquisition technologies presented in each IOS device. According to the manufacturer, CS 3600 is a structured LED light Scanner that captures images according to the principle of the active speed 3D video. It employs the Intelligent Matching System, which allows the software to connect the scanned images very quickly and build the mesh continuously, without interruption. On the other hand, Trios 3 is a structured light scanner that uses confocal microscopy and Ultrafast Optical Scanning technology to capture more than 3000 two‐dimensional images per second. It is obvious that different IOS technologies affect quality parameters, such as trueness and precision, while further investigation is necessary to reveal the exact impact on clinical relevance.

A notable distinction between the two clinical scenarios simulated in this study was the predominant occurrence of angular deviations in the first scenario, while both angular and linear deviations were observed in the second scenario. Additionally, the second scenario exhibited higher angular and linear deviations for anterior implants (lateral incisors). This may be attributed to the 20° buccal angulation of these implants. Previous literature suggested that implant angulation exceeding 15° may have a detrimental impact on digital impression accuracy [20, 27]. Moreover, Ebeid et al. [34] also concluded that angulated implants negatively impact scanning accuracy [34]. The authors investigated various laboratory scanners for fabricating full‐arch dental prostheses with different implant angulations, and found that only trueness but not precision was affected by implant angulations. The difference was attributed to the stability of the scanned casts during extraoral scanning, a process that involves utilizing a fixed scanner rather than a handheld intraoral scanner [34]. On the contrary, there are still other studies suggest that implant angulation does not significantly affect impression quality [17, 35, 36].

The issue of implant angulation is of significant concern, as bone deficiencies often impede implant distribution and angulation in edentulous arches, potentially compromising osseointegration. A recent in vitro study compared the accuracy of implant impressions obtained using computer‐aided impression‐making technology and a conventional approach when implants were angled between 40° and 45° [34]. The findings revealed that tilted implants exhibited reduced accuracy compared to parallel implants, while both digital and conventional impression‐making techniques produced comparable results across all case scenarios. Furthermore, Moslemion et al. [37] examined the impact of scan body types and shapes on digital impression accuracy and scanning time [37]. Intriguingly, the authors discovered that implant angulation differentially influenced accuracy depending on the connection and scan body type employed. These findings emphasize the significance of implant angulation during the scanning process, suggesting that a degree‐based angulation threshold should be established by researchers to prevent errors.

Accurate capture of implant distances in complete arch impressions remains a key concern. Spagopoulos et al. [38] evaluated in vitro the accuracy of various IOS and laboratory scanners in a 4‐implant model, assessing the impact of implant spacing on scanner trueness and precision. They concluded that laboratory scanners had better trueness and precision than all IOSs for long distances and that most IOSs were more reliable in smaller intra‐arch spans [38]. However, our study observed comparable accuracy for both scanner types in full‐arch implant acquisitions. Further studies are needed to assess the impact of implant distance on the accuracy of digital impressions.

The present study employed an in vitro protocol, which represents a limitation as it does not fully replicate the complexities of the in vivo environment. Intraoral scanning may encounter challenges, such as tissue undercuts, limited space, saliva, gag reflex, patient movements, and the inability of the scanner tip to reach posterior regions, all of which can potentially impact the accuracy of digital impressions [39, 40]. Furthermore, all scans in the present study were performed by a single operator. The experience of the operator has been shown to have an impact on accuracy in some studies [32], while in others no significant influence was reported [19]. Additional scans from other dental investigators could be an asset to validate results in further investigations. Moreover, different scanning strategies may lead to varying results. This study utilized the manufacturer's recommended scanning strategy. Different strategies have been employed in literature, while the impact of these strategies on digital impression accuracy in fully edentulous arches remains incompletely understood [41]. Future research should investigate the influence of the scanning protocol, as well as other factors affecting scan quality, such as implant angulation or splinting of the abutments to improve scanning outcomes.

## 7 Conclusion

Two IOS devices were evaluated for accuracy in acquisition of digital impressions of implant‐supported all‐on six maxillary restorations. Both scanners provided clinically acceptable accuracy, with Trios demonstrating superior precision and Carestream exhibiting superior trueness. Digital impressions are increasingly becoming an essential part of daily clinical practice. Implant angulation and scanning protocol play a significant role in determining the quality of digital acquisitions, especially in the most challenging clinical scenarios, such as full‐arch implant‐supported prostheses with angulated implants in both anterior and posterior areas of the maxilla.

## Conflicts of Interest

The authors declare no conflicts of interest.

## Data Availability

The data that support the findings of this study are available from the corresponding author upon reasonable request.
